# Bovine Tuberculosis Surveillance System Evaluation, Greater-Accra Region, Ghana, 2006-2011

**DOI:** 10.11604/pamj.supp.2016.25.1.6181

**Published:** 2016-10-01

**Authors:** Perdita Hilary Lopes, Patricia Akweongo, Fred Wurapa, Edwin Afari, Samuel Sackey, Dennis Ocansey, Kofi Mensah Nyarko

**Affiliations:** 1 Veterinary Services Directorate, Accra, Ghana; 2 Ghana Field Epidemiology and Laboratory Training Programme, School of Public Health, University of Ghana, Legon; 3 Epidemiology Department, School of Public Health, University of Ghana, Legon; 4 Ghana Health, Service, Accra, Ghana

**Keywords:** Bovine tuberculosis, Mycobacterium bovis, surveillance, evaluation, Ghana

## Abstract

**Introduction:**

Bovine tuberculosis (bTB) is a chronic, zoonotic, multi-species disease of cattle caused by Mycobacterium bovis. In developed countries, effective surveillance and enforcement of regulations on bTB control resulted in significant reduction of infections in cattle and hence, humans. However, in developing countries, weak surveillance systems affect accurate and timely reporting of bTB in humans and cattle. In Ghana, transhumance movement of cattle increases the risk of bTB importation and spread, however, the extent to which surveillance detects bTB is unknown. We therefore evaluated the bTB surveillance system in the Greater-Accra Region to determine its performance and assessed its attributes.

**Methods:**

We interviewed stakeholders, and reviewed bTB surveillance data for all ten districts in the region from 2006-2011 using the CDC Guidelines for Evaluation of public health surveillance systems.

**Results:**

From 2006-2011, bTB was suspected in 284/244,576 (0.12%) cattle slaughtered, of which 7/284 (2.5%) were submitted for laboratory confirmation and all tested positive. Predictive value positive was 100%. There is no standard case definition which guides bTB detection. Fifty percent of carcasses slip through inspection, and confirmed cases are not traced back. There were 99/284 (34.9%) condemnations from suspected carcasses and 57/97 (58.8%) from positive reactors from screening. Ninety percent (9/10) of districts submitted reports late to the region whereas representativeness was 30%. Regional and district data were manually stored with no electronic backups. The region's cattle population is unknown.

**Conclusion:**

Although the bTB surveillance system is sensitive, it is under performing, and the possibility of bTB transmission from cattle to humans is high.

## Introduction

Bovine tuberculosis (bTB) is a debilitating, chronic disease of cattle caused by Mycobacterium bovis from the Mycobacterium tuberculosis complex of bacteria. The bacterium is ubiquitous and has worldwide distribution, however, developing countries are most affected [1–3]. Bovine TB affects other species, including man, and causes reduced productivity in livestock [3–6]. Additionally, bTB infections in humans are very difficult to differentiate clinically from infections due to M. tuberculosis [7, 8]. Persons with weak or underdeveloped immune systems such as HIV patients, the young and elderly are particularly vulnerable to Mycobacteria infections which includes infections with M. bovis [9–11]. This, arguably, means treatment is often protracted leading to increased morbidity and mortality as a result of drug resistance with huge financial implications. This situation is particularly dangerous considering that the burden of both HIV and tuberculosis is very high in Africa [11–13]. For these reasons, huge national funds are spent on bTB control [14, 15]. Low bTB prevalence in developed countries has been attributed to effective surveillance activities which included detection of bTB during meat inspection, trace back of carcasses which are bTB suspected, restriction of movement from infected herds, and awareness creation on its economic and health implications [16–18]. These, together with the enforcement of regulations such as condemnation of bTB infected carcasses or organs, periodic test and slaughter or segregation, pasteurization of milk and restriction of breeding from infected herds, culminated in the reduction of human TB infections due to M. bovis from 5%-20% to 0.5%-1% [19–22]. However, in Africa where bTB surveillance is either weak or non-existent, the contribution of M. bovis to human tuberculosis infections is higher; 10-15%, with some individual countries reporting as high as 50% herd prevalence [2, 19, 23]. The World Organization for Animal Health (OIE) has classified bTB as a list B disease due to its economic and public health importance, therefore the ultimate goal of surveillance is to achieve freedom from it and eventually, eradicate it [24]. In order for any country to be certified free from bTB however, bovines, water buffalo and bison populations must meet OIE requirements with respect to their bTB status [24]. Three of these criteria, which Ghana already satisfies, requires that bTB infection in cattle must be a notifiable disease in the country, surveillance must be maintained to detect bTB through meat inspection, and there must be an ongoing awareness programme to encourage reporting of all cases suggestive of bTB. Additionally, regular and periodic testing of all cattle should have demonstrated absence of M. bovis infection in at least 99.8% of the herds and 99.9% of the cattle in the country for three consecutive years [24]. In Ghana, the bovine species available are cattle; hence these conditions will pertain to them. Multiple borders and low cattle populations means that transhumance cattle movement is high from neighbouring countries with limited tracking. The potential to import bTB is therefore high. Ghana's policy on bTB surveillance includes restriction of movement and breeding from cattle infected with bTB. However, there is weak enforcement of these policies. The extent to which the bTB surveillance system is meeting its objectives is thus unknown as it has not been evaluated to assess its performance for over a decade. This study therefore seeks to evaluate the bTB surveillance system in Greater-Accra, the nation's capital in order to assess whether the surveillance system is achieving its objectives, and also to assess standard surveillance system attributes using the Centres for Disease Control updated guidelines for public health surveillance systems.

## Methods

Study area, design and sources of data: the study area was the Greater-Accra Region of Ghana which is located in the south-east of the country along the Gulf of Guinea, between latitude 50 330 North, 00 130 West. It occupies a land surface area of 3,245 square kilometres, with an estimated population of 4,010,054 [25] and comprises of ten districts. The region's cattle population is unknown. However, the cattle population in the country is estimated at 1.5 million [26]. We reviewed veterinary monthly reports for all ten districts in the region, as well as screening and laboratory records of bTB tests from January 2006-December 2011. We interviewed stakeholders at national, regional and district levels. These included the Director of Veterinary Services, the National Veterinary Epidemiologist and Public Health Officers, the Regional and District Veterinary Officers and their staff, as well as butchers and cattle merchants. We evaluated the operation of the system with respect to case definitions, resources and reporting. Attributes assessed include sensitivity, usefulness, stability, acceptability, representativeness, completeness, timeliness and data quality. Results were expressed as frequencies, percentages, ranges and means. Graphs were drawn to show trend. Permission was obtained from the office of the Director of the Veterinary Services Directorate (VSD) to access official documents on bTB, and the School of Public Health, University of Ghana, Legon.

## Results

**Case definitions:** there was no official written case definition for suspected and probable cases of bTB. However, its suspicion and detection during meat inspection was based on the identification of granulomatous lesions in the lungs, intestines and other affected organs. All stakeholders interviewed were familiar with bTB infected carcasses and described bTB lesions as whitish granular nodules, which “sounded like a knife cutting through sand upon incision”. Similarly, animals in contact with suspected or confirmed cases were referred to as probable cases, whereas confirmed cases were referred to as samples testing positive to Ziel-Neelson's acid fast stain test for tuberculosis.

**Cattle population:** the number of cattle herds and cattle in each district, and the region for that matter, is unknown.

**Figure 1:**
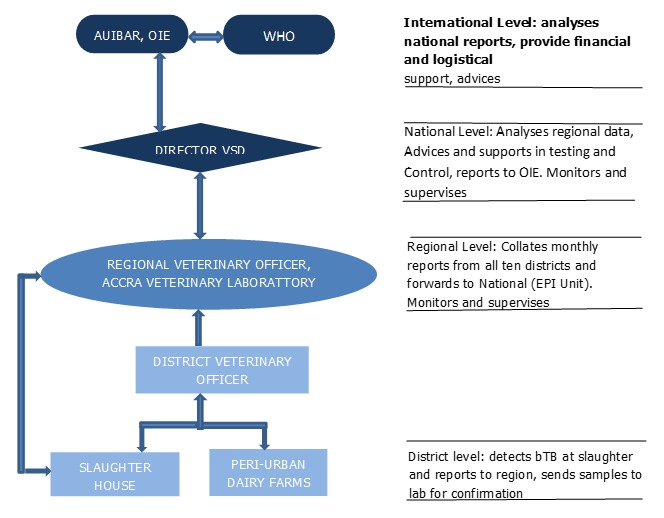
Flowchart of Bovine tuberculosis surveillance system, Greater Accra, Ghana

**Morbidity and mortality:** there was an upward trend in bTB suspicion during slaughter from 2006-2008, but this took a dip in 2009, rose slightly in 2010 and dipped again in 2011 as shown in Figure 1.

**Figure 2:**
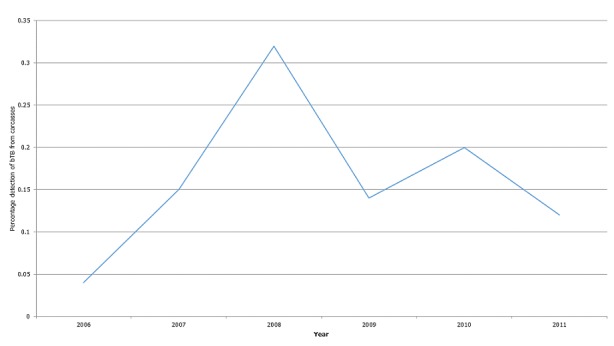
Percentage and trend of bTB in carcasses, Tema Metropolitan Area, 2006-2011

**Data flow:** the flow of data in the bTB surveillance system in the Region is shown in Figure 2. Farmers and veterinary technical officers who suspect bTB in a herd report same to the District Veterinarian who follows up and reports his oe her findings to the Regional Veterinarian through monthly reports. Bovine TB detected during routine meat inspection is also documented and reported to the Region at the end of the month since the disease is not an immediately notifiable one. Carcasses in which bTB lesions are suspected are either partially or totally condemned depending on how widespread the lesions are. Ideally, District Veterinary Officers are required to conduct an epidemiological trace back to the herd from which the infected animal originated, but no such evidence was found. The Regional Veterinarian collates reports from all ten districts and forwards them to the national level where an epidemiologist analyses all the data. This is then forwarded to the OIE and the African Union Inter-African Bureau Resource (AU-IBAR).

**Resources for bTB surveillance:** The cost of bTB surveillance is covered under the budget allocation for scheduled diseases surveillance. On average $1,667.00, $500.00 and $444.00 respectively is allocated to the national, regional and district levels every quarter for disease surveillance and control activities including bTB. However, in 6/10 (60%) of the districts evaluated, these funds were sometimes not made available for that purpose by the District Directors of Agriculture. They were rather used for other administrative activities [27].

**Types of surveillance:** two main types of bTB surveillance were observed, namely, active and passive. Active surveillance comprised detection during meat inspection, targeted screening of cattle in an area based on detection of more than five bTB cases from that particular area within a month, and laboratory testing and confirmation using Ziehl-Neelsen staining technique. There was no systematic routine screening in place, but screening upon request in farms that were mainly public occurred. Passive surveillance was based on voluntary reporting by peri-urban dairy farms and cattle herdsmen to district veterinary staff. However, bTB detection by this method was rare. Out of nine such reports, only one (1/9) farmer reported suspicion of bTB in a cow slaughtered for domestic consumption. Otherwise all the remaining 88.88% (8/9) voluntary reports came from public farms.

**Documentation and data quality:** the Accra Veterinary Laboratory records all animal and carcass sample test results for bTB, however, documentation was not uniform. Results were recorded as AFB, positive, Acid Fast Bacilli, TB positive, MTB, some were scored, whereas others were not. Notwithstanding this, there was clear client and specimen identification, and detailed information regarding receipt and processing of samples at the laboratory. Results were entered into a log book, and correspondence on bTB stored in a file.

**Attributes of the bTB surveillance system. Sensitivity, predictive value positive and usefulness:** the surveillance system was found to be sensitive as it was able to detect cases of bTB. The number of cattle slaughtered from 2006-2011 was 44,460, of which 284 (0.12%) were suspected to be bTB infected. Of these, 7/284 (2.46%) were sent to the laboratory for confirmation and all tested positive (Predictive Value Positive = 100%). The number of cattle which tested positive out of 3,367 screened was 97 (2.9%). The usefulness of the surveillance system was found to be low; out of the 284 carcasses suspected to be bTB infected, there were 99 (32.04 %) total and partial condemnations; 8/99 (8.08%) of these were totally condemned. Similarly, out of the of 97 animals detected to be bTB infected from screening, 57 (58.76%) were culled, as shown in Table 1. However, bTB was neither detected in any of the 1,113,603 cattle moved across the region from one location to the other for breeding, nor did movement permits certify whether these cattle originated from bTB free herds. Finally, whereas public farms routinely pasteurized their milk, and occasionally tested for bTB, there was no surveillance on the status of milk from private cattle farms with respect to bTB.

**Table 1:** Sensitivity, Predictive Value Positive and Usefulness of the bTB Surveillance System, Greater-Accra region, 2006-2011

	Activity			
	Meat Inspection	Screening	Laboratory Confirmation	Movement of Livestock
No of animals	244,460	3,367	7	113,603
Number Detected/Confirmed	284	97	7	o
% Detection	0.12	2.8	100	nil
Total Condemnations	8	57	1	nil
Partial Condemnations	91	nil	Nil	nil

**Simplicity:** the bTB surveillance system was found to be complicated in structure as detection requires special training, particularly in the screening of animals and laboratory confirmation of the disease. Also, only the Accra Veterinary Laboratory, which is at the regional capital, has the facilities to test for bTB in the region, as such, all suspected samples should be transported for confirmation. This situation was further complicated by the lack of transportation to send samples.

**Acceptability:** twelve percent (3/25) of stakeholders interviewed admitted willingness to comply with bTB reporting. Butchers and cattle merchants explained that a disincentive for voluntary reporting of suspected bTB cases was the lack of compensation for cattle farmers. This is because animals which are condemned on suspicion of bTB imply financial loss to the farmer.

**Stability:** the system was found to be unstable for the following reasons: at regional and district levels, surveillance data were stored manually or on personal computers of staff, and some monthly reports could not be traced. Additionally, most districts had inadequate staff who were poorly resourced to keep surveillance on scheduled diseases including bTB. This was exacerbated by irregular release of surveillance funds.

**Flexibility:** At the district level, bTB suspected carcasses at slaughter were reported on Veterinary Form 9 which is also used to capture other diseases at the end of the month. Hence the system was flexible. However, there were no daily records of slaughter findings.

**Representativeness and completeness:** three districts out of ten (30%), reported on bTB in their monthly reports, and one district did this consistently. Moreover, not all cattle were slaughtered under veterinary supervision; hence some incidence of bTB may have been missed in official reports. In all districts, Veterinary Form 9 was missing for some of the months, making reporting incomplete.

**Timeliness:** one district out of ten (10%) regularly submitted monthly reports on time. Delays ranged from 1-26 days. In contrast, reports from laboratory investigations of samples submitted for bTB testing were released to clients in a timely manner, on the same day.

**Data analysis:** data was neither analysed at the district nor regional levels. They were merely compiled and forwarded to the Epidemiology Unit, which performed the analysis. There was feedback from laboratory testing to the districts; however, feedback from the regional veterinary office to the districts was absent.

## Discussion

**Statement of Principal Findings:** the bTB surveillance system of the Greater-Accra Region is able to detect infection both in live animals and carcasses; however, there was no officially written case definition for bTB. Diagnosis, confirmation and screening were all based on the presence of lesions or reactions which are characteristic for bTB infected carcasses or animals; therefore all the criteria used were acceptable. However, this is in contrast to human tuberculosis which has well defined case definitions as found in the Integrated Disease Surveillance and Response System of Ghana [27]. The prevalence of bTB suspected carcasses at slaughter was 0.12%. This is similar to that found by a study in the highlands of Cameroun, and Maiduguri in Nigeria [28, 29], but in sharp contrast to other studies in Kenya and Ethiopia where the prevalence was much higher; 18.5% and 28.2% respectively [30, 31]. The majority of bTB detections were made at slaughter, however, studies have demonstrated that not all cattle infected with bTB may present with macro lesions which can be detected during post mortem inspection [32–34]. Therefore the number of bTB infected carcases detected by this method could just as well be the tip of the iceberg. All these studies suggest that post mortem inspection should therefore be consolidated with ante-mortem findings and laboratory confirmation for carcasses from bTB suspected animals during ante mortem inspection, but which may not show macro lesions during post mortem inspection, as is done in the US [16]. Additionally, since no daily records were kept of slaughter activities and findings, it was unclear how monthly estimates of suspected cases were arrived at. This compromises the reliability of the number of bTB cases reported. On the other hand, butchers and meat inspectors interviewed revealed that a significant number of carcasses were not subjected to post mortem inspection due to lack of cooperation on the part of some butchers. They estimated that of the number of carcasses that are subjected to post mortem inspection, an equal number slip through without inspection. Furthermore, not all the suspected cases are reported. All these grossly undermine the sensitivity of this method for the detection of bTB. The possible reasons for the refusal of butchers to comply with reporting may be due to ignorance about the health implications of bTB, but perhaps most importantly, lack of compensation. A study in Switzerland found that increasing awareness about the economic and public health implications of bTB increases its reporting significantly [35]. Additionally, it appears the slaughter of positive reactors was left solely to the discretion of owners who were often unwilling to comply. In order to increase acceptability, some countries encouraged voluntary reporting and compliance with compulsory slaughter of positive reactors through farmer compensation. For example in Egypt, farmer compensation resulted in the reduction of bTB from 6.2%-9.4% in two governorates to about 2.6% between 1981-1985 [36–38]. Furthermore, the number of samples which were sent for laboratory confirmation was rather low. This was attributed to lack of transportation to convey samples to the laboratory. From screening exercises, the prevalence of bTB in animals tested was 2.9%, in sharp contrast to 14.3% in Pradesh in India, and 14% in Nigeria [39, 40]. This may be due to the fact that the proportion of cattle screened during the period under review was also low, hence missing more bTB infected herds or cattle.

The usefulness of the surveillance system was found to be very low; only 32% of infected carcasses were either totally or partially condemned, whereas 58.7% of positive reactors from screening were culled. It is unclear what was done to the remaining infected carcasses. There was also no evidence to indicate that epidemiological trace back was done to identify the herds from which these animals originated. This could have been due to lack of a proper cattle identification system in place in the country. In a study in England on post slaughter detection of bTB in carcasses, improper identification of animals was found to be a major factor contributing to the inability to trace back infected carcasses to their herds [41]. This means potentially infected herds may have been missed. Additionally, it appears that screening was limited to a few public farms over the years. There was no evidence as to whether the herds were placed under quarantine or re tested as recommended by the Guidelines for the control of Bovine Tuberculosis [42]. Further, in order to make scientific estimates of bTB prevalence in any given zone, and to assess whether disease control targets are being met, a certain percentage of cattle herds and cattle must be screened [23]. There is therefore the need for a systematic, random testing of cattle herds based on reliable surveillance data to determine districts at risk. Since there was neither data on the number of cattle herds nor cattle population in the region, there is no denominator data to determine if the region was on course to controlling bTB. The rise in bTB detections from 2006-2009 could be attributed to the arrival of a new district veterinarian, and this is supported by a dip in reporting during his brief absence in 2009. This clearly indicates that not all staff are mounting optimum surveillance on bTB in the region. Considering the absence of epidemiological trace back of carcasses from which bTB was suspected, it implies that other potentially tuberculous cattle or herds could not be identified for further investigation. Consequently, such animals could also end up in the food chain or be moved from one location to the other, further spreading the disease. Similarly, animals which were moved for breeding purposes were given a general visual health examination which did not include certification of bTB status. Thus there is no guarantee that they originated from bTB free herds, which is one of the objectives of bTB control in Ghana. One of the methods used to control the spread of bTB in bTB free states such as Australia and the US was quarantine and control of movement of cattle from infected herds [16, 42]. It is worth noting that, apart from public farms, milk from all private cattle farms visited was sold directly to consumers without being pasteurized. This could potentially be hazardous considering that the status of such farms with respect to bTB is unknown. In developed countries, one of the recommended and successful methods applied in the control of bTB transmission to humans was through pasteurization of milk [43]. Resources for bTB surveillance are woefully inadequate as there is no specific budget line for bTB surveillance; rather, the general budget for diseases surveillance is used. The flow of funds is also unreliable, hence impacts negatively on the system's stability. This is not surprising considering that health systems, and disease surveillance for that matter, are generally poorly financed in Africa [44–46]. This is in sharp contrast to the situation in developed countries where disease surveillance systems are well funded to ensure timely detection and control.

**Strengths and weaknesses of the study:** this study has clearly demonstrated the areas where there are lapses in the bTB surveillance system, however, it was unable to establish by how much bTB detection is being missed. A more detailed study would be required to investigate the actual prevalence of bTB at slaughter, national herd prevalence and the bTB status of cattle moved for breeding purposes. The latter, in turn would depend on an effective cattle identification program, which currently, is lacking in Ghana.

## Conclusion

Although the bTB surveillance system is sensitive and flexible, reporting is neither complete nor representative of the actual situation on the ground, timeliness is poor and there is generally no feedback from the region to the districts. Usefulness of the methods applied in bTB surveillance were found to be low, as was acceptability. The system was found to be unstable and data quality was poor, and the availability of resources for bTB surveillance untimely and woefully inadequate. Additionally, denominator data is lacking; actual cattle populations in the ten districts remain largely unknown. Therefore the BTB surveillance system is achieving some of its objectives; however, there is room for improvement. The possibility of bTB transmission from cattle to humans is high. The Ministry of Food and Agriculture should invest more resources to support bTB surveillance and the implementation of its control as this will go a long way to reduce transmission of the disease to humans. This should include compensation of farmers whose animals are detected to have bTB as this will improve acceptability. The VSD should increase bTB awareness creation among farmers and other key stakeholders. It should also train all veterinary officers on the importance of bTB surveillance in order to improve on representativeness, timeliness and completeness of reporting. All district officers should compile a database of cattle farms in their district for easier surveillance. Animals which are moved for breeding purposes should be certified to originate from bTB free herds. The head of the Epidemiology Unit has since drafted case definitions not only for bTB but also for all other scheduled diseases. Districts which were formerly not reporting on bTB surveillance have started doing so in their monthly reports following training on the importance of bTB reporting. Discussions have been held with the Director, VSD and plans are under way to organize bTB awareness creation in collaboration with the Ghana Health Service, Butchers Association and other key stakeholders.
